# Textilinin-1, a Snake Venom-Derived Kunitz-Type Protease Inhibitor, Accelerates Wound Healing Through Anti-Inflammatory, Antibacterial, and Pro-Regenerative Activities

**DOI:** 10.3390/pharmaceutics18060762

**Published:** 2026-06-22

**Authors:** Zhuo Chen, Huiwen Pang, Youzhi Wu, David M. Klyne, Xuqiang Nie, Pengfei Jiang, Xinfei Wu, Kong-Nan Zhao, Felicity Y. Han

**Affiliations:** 1 Australian Institute for Bioengineering and Nanotechnology, The University of Queensland, Brisbane, QLD 4072, Australia; zhuo.chen6@student.uq.edu.au (Z.C.);; 2 Centre for Innovation in Pain and Health Research (CIPHeR), School of Health and Rehabilitation Sciences, The University of Queensland, Brisbane, QLD 4072, Australia; d.klyne@uq.edu.au; 3 Key Lab of the Basic Pharmacology of the Ministry of Education & Joint International Research Laboratory of Ethnomedicine of Ministry of Education, Zunyi Medical University, Zunyi 563006, China; niexuqiang@zmu.edu.cn; 4 Institute of Molecular Virology and Immunology, Department of Microbiology & Immunology, School of Basic Medical Sciences, Wenzhou Medical University, Wenzhou 325035, Chinacervicalcancer@wmu.edu.cn (X.W.)

**Keywords:** Textilinin-1, wound healing, anti-inflammatory, proliferation

## Abstract

**Background/Objectives**: Chronic wounds remain a formidable clinical challenge due to the suboptimal efficacy of conventional delivery systems and therapeutics. Textilinin-1, a venom-derived Kunitz-type serine protease inhibitor, has previously established its profile as a potent hemostatic agent. However, its potential as a multifunctional biopharmaceutical for wound management remains largely untapped. This study evaluates the pharmacological effects of Textilinin-1 in preclinical models of cutaneous wound repair. **Methods**: We employed an integrated platform comprising bioinformatics, in vitro cellular assays, and in vivo murine excisional wounds and a pilot porcine proof-of-concept model to assess the wound healing-promoting effects of Textilinin-1 and explore associated cellular responses associated with key stages of the wound healing cascade. **Results**: Textilinin-1 was associated with multiple cellular responses relevant to tissue repair. It attenuated M1-like inflammatory activation and showed preliminary growth-inhibitory activity against *Staphylococcus aureus* under the tested conditions. Concurrently, it enhanced the proliferative and migratory capacity of fibroblasts, endothelial cells, and keratinocytes, which are key cellular targets for wound closure. In pre-clinical pilot porcine and rodent models, Textilinin-1 treatment was associated with accelerated wound contraction and improved structural tissue quality. **Conclusions**: Our findings provide preclinical evidence that Textilinin-1 may promote cutaneous wound repair and modulate cellular responses relevant to key stages of the wound healing cascade. These results support further investigation of Textilinin-1 as a candidate for wound repair applications. Future studies are required to define its precise molecular mechanisms, evaluate its efficacy in chronic or otherwise compromised wound models, and optimize its topical formulation or hydrogel-based delivery.

## 1. Introduction

Wound healing is a coordinated multicellular process, spanning four overlapping stages: haemostasis, inflammation, proliferation, and remodelling [1,2,3]. While this sequence preserves cutaneous barrier integrity under normal conditions, various factors and comorbidities can derail it, leading to ongoing chronic wounds that substantially burden patients and healthcare systems [4,5,6,7]. Globally, nearly 1 billion people are affected with associated costs up to ~3% of total health expenditures in developed nations [8,9]. Unfortunately, current therapies, including debridement and antimicrobials, advanced dressings, biologicals, and other emerging technologies, show limited efficacy and durability across diverse patient subgroups and wound types, underscoring the urgent for new treatments based on a clearer understanding of healing mechanisms [7,10,11,12,13].

Animal venoms, particularly from snakes have emerged as rich sources of pharmacological leads due to the high specificity and potency of their bioactive components [14]. Textilinin-1, a 6.7 kDa, a Kunitz-type serine protease inhibitor isolated from Pseudonaja textilis (*P. textilis*) venom, is a potent and selective inhibitor of plasmin and trypsin [6,15]. It demonstrates superior selectivity compared to aprotinin, an inhibitor that acts on a wide range of proteases [16,17]. This selectivity allows Textilinin-1 to avoid key off-target enzymes; for example, it only weakly inhibits plasma kallikrein (Ki ≈ 4.8 μM), whereas aprotinin inhibits this enzyme potently (Ki* ≈ 16.4 nM) [16,17]. This distinction is critical, as strong inhibition of kallikrein by aprotinin has been implicated in adverse clinical events [16,17]. Preclinically, recombinant Textilinin-1 (Q8008) has demonstrated anti-bleeding efficacy comparable or superior to aprotinin, but with a faster onset of action and an improved safety profile.

Haemostasis is not merely a plug to stop bleeding, it provides the provisional matrix that scaffolds the subsequent inflammatory and proliferative phases of repair [1,2,3]. Since serine proteases like plasmin play dual roles in both fibrinolysis and the modulation of cellular migration and tissue remodeling, inhibitors like Textilinin-1 may possess therapeutic potential beyond simple haemostasis [16,17]. However, while the antifibrinolytic activity of Textilinin-1 is well characterized, its effects on wound healing-related cellular responses on the cellular mechanisms of tissue regeneration remains unexplored [16,17]. Given that plasmin and related trypsin-like serine proteases are involved in fibrinolysis, inflammatory regulation, extracellular matrix turnover, cell migration, and protease-activated receptor signalling [18], we investigated the potential of Textilinin-1 to influence key cellular processes in wound healing, including immune modulation, proliferation, migration, remodelling, and pro-angiogenic responses. Using an integrated approach, including in silico bioinformatics protein–protein interaction (PPI) network analysis, in vitro assays in key wound cells (macrophages, fibroblasts, endothelial cells, and keratinocytes), in vivo testing in a murine and pilot pig wound model and exploratory protein-protein docking.

## 2. Materials and Methods

### 2.1. Materials

Recombinant Textilinin-1 (Q8008) with the same amino acid sequence as native Textilinin-1 was provided by AIBN Venomics Group (Kong-Nan Zhao and Martin Lavin) [19], The University of Queensland. Dulbecco’s Modified Eagle Medium (DMEM; high-glucose 4.5 g/L and low-glucose 1 g/L), keratinocyte basal medium (KC-SFM, 0.09 mM Ca^2+^), fetal bovine serum (FBS), L-glutamine, phosphate-buffered saline (PBS), and trypsin–EDTA were obtained from Gibco (Thermo Fisher Scientific, Grand Island, NY, USA). Lipopolysaccharide (LPS; from *E. coli* O111:B4), hydrogen peroxide (H_2_O_2_), crystal violet, and paraformaldehyde (PFA) were purchased from Merck (Burlington, MA, USA). The Cell Counting Kit-8 (CCK-8) was obtained from Abcam (Waltham, MA, USA).

Antibodies for flow cytometry, including PE-conjugated anti-mouse ARG1 and Alexa Fluor^®^ 647-conjugated anti-iNOS, as well as fixation/permeabilization reagents, were purchased from BioLegend (San Diego, CA, USA). The fluorescent probe 2′,7′-dichlorofluorescein diacetate (DCFH-DA) was from Sigma-Aldrich (St. Louis, MO, USA). EHS matrix extract and Transwell inserts (8 μm pore size) were obtained from Merck (Boston, MA, USA).

The NIH/3T3 (mouse fibroblast), RAW 264.7 (mouse macrophage), and SVEC4-10 (mouse endothelial) cell lines were used in this study. Human primary keratinocytes (provided by the Saunders group) [20] and normal human dermal fibroblasts (NFFs, PromoCell GmbH, Sickingenstr, Heidelberg, Germany) were isolated from foreskin tissue and cultured as described previously [21,22]. Unless otherwise specified, all other reagents were of analytical grade and obtained from standard commercial sources.

### 2.2. Bioinformatics Prediction

The amino acid sequence of Textilinin-1 (UniProt ID: Q90WA1) was retrieved from the UniProt database [23]. Potential molecular targets were identified based on two established functional targets of Textilinin-1, Plasmin (PLG) and Trypsin (PRSS1), through PPI analysis using the STRING database [24], which integrates sequence similarity with experimentally validated protein interactions. To ensure analytical robustness, only interactions with a confidence score ≥0.7 (high confidence) were included. A comprehensive list of wound healing–associated genes was obtained from the GeneCards database [25] using the keyword “wound healing” This gene set was subsequently cross-referenced with the PPI networks for PLG and PRSS1 via the Venn Database (https://bioinformatics.psb.ugent.be/webtools/Venn/) (accessed on 4 February 2025) to identify overlapping genes, representing potential mediators of Textilinin-1 activity. These intersecting genes were further analyzed in STRING to construct a high-confidence, integrated PPI network, which was visualized using Cytoscape (v3.9.1) [26]. Functional annotation of the PPI network was performed through Gene Ontology (GO) and Kyoto Encyclopedia of Genes and Genomes (KEGG) enrichment analyses using the DAVID bioinformatics platform [27], with enrichment results visualized via the Bioinformatics online platform [28].

### 2.3. Cell Culture

The NIH/3T3, RAW 264.7, and SVEC4-10 cell lines used in this study were authenticated by their respective providers based on cell size, karyotype, and morphological characteristics, and confirmed to be free from contamination. Human primary keratinocytes isolated from the foreskin were cultured in vitro as described previously [21]. Normal human dermal fibroblasts (NFF) isolated from foreskin were cultured in vitro as described previously [22]. Keratinocytes were cultured in KC-SFM medium with low calcium (0.09 mM). NFF cells were maintained in Dulbecco’s Modified Eagle Medium (DMEM) supplemented with 10% fetal bovine serum (FBS) and 2 mM L-glutamine. The SVEC4-10 cell line was cultured in low-glucose DMEM (1 g/L glucose), whereas NIH/3T3 and RAW 264.7 cells were cultured in high-glucose DMEM (4.5 g/L glucose). All media were supplemented with 10% FBS unless otherwise stated. Unless specified otherwise, all cell culture reagents were obtained from Gibco (Thermo Fisher Scientific, Grand Island, NY, USA). Cells were maintained at 37 °C in a humidified incubator with 5% CO_2_. For induction of the in vitro inflammatory model, RAW 264.7 cells were stimulated with 100 ng/mL lipopolysaccharide (LPS) (Burlington, Merck, MA, USA) for 24 h before treatment with vehicle or 8 μM Textilinin-1.

### 2.4. Flow Cytometry

In RAW 264.7 cells, experiments were conducted to assess macrophage polarization and the anti-inflammatory effects of Textilinin-1 by quantifying the expression of inducible nitric oxide synthase (iNOS; an M1 phenotype marker) and arginase-1 (ARG1; an M2 phenotype marker) via flow cytometry [29]. Briefly, RAW 264.7 cells were seeded into 6-well plates at a density of 3 × 10^6^ cells/well and incubated overnight in standard culture medium. Cells were then allocated to one of four treatment groups: control (untreated), Textilinin-1 (8 μM Textilinin-1), LPS (100 ng/mL LPS), or LPS + Textilinin-1 (100 ng/mL LPS + 8 μM Textilinin-1). For the LPS and LPS + Textilinin-1 groups, cells were first stimulated with 100 ng/mL LPS for 24 h. During the following 24 h, the control and LPS groups received vehicle, whereas the Textilinin-1 and LPS + Textilinin-1 groups received 8 μM Textilinin-1. All groups were maintained for the same total experimental duration before analysis. At the end of treatment, cells were gently scraped, collected, and washed three times with cold phosphate-buffered saline (PBS). For intracellular staining, cells were fixed and permeabilized with 250 μL fixation/permeabilization solution (BioLegend, San Diego, CA, USA) at 4 °C for 20 min. Following two washes in permeabilization buffer, cells were incubated at 4 °C for 30 min in the dark with PE-conjugated anti-mouse ARG1 antibody (0.125 μg per 1 × 10^6^ cells in 100 μL) and Alexa Fluor^®^ 647-conjugated anti-Nos2 (iNOS) antibody (0.25 μg per 1 × 10^6^ cells in 100 μL) (both from BioLegend, San Diego, CA, USA). After staining, cells were washed, resuspended in PBS, and analyzed on a CytoFLEX flow cytometer (Beckman Coulter, Brea, CA, USA). Fluorescence was acquired using the PE and APC channels for ARG1 and iNOS, respectively. Data were processed and quantified with FlowJo software (BD Biosciences, Ashland, OR, USA). All experiments were independently repeated three times to ensure reproducibility.

### 2.5. Antibacterial Activity Assay

The Gram-negative bacterium Escherichia coli (*E. coli*) and the Gram-positive bacterium Staphylococcus aureus (*S. aureus*) were selected as representative strains to evaluate the antibacterial activity of the test samples [30,31]. All bacterial strains were stored at −80 °C in LB broth (BD Biosciences, USA) supplemented with 20% (*v*/*v*) glycerol. For revival, 20 µL of frozen bacterial stock was inoculated into 5 mL of fresh LB broth, mixed thoroughly, and incubated overnight at 37 °C with shaking at 180 rpm. Overnight cultures were diluted in fresh LB broth to an initial optical density at 600 nm (OD_600_) of 0.05. Test samples were added to the bacterial suspensions at the indicated final concentrations. Cultures were incubated at 37 °C with continuous shaking, and bacterial growth was monitored by measuring OD_600_ at predetermined time points (0, 2, 4, 8, and 24 h) using a microplate reader (Infinite 200 PRO, TECAN, Männedorf, Switzerland). Growth curves were generated by plotting OD_600_ values against incubation time. To visually assess bacterial survival, a colony counting assay was performed. Bacterial suspensions from each group were collected after 12 h of incubation and serially diluted 10-fold in fresh LB broth (BD Biosciences, USA). Aliquots (50 µL) from each dilution were spread evenly onto LB agar (BD Biosciences, USA) plates in triplicate and incubated at 37 °C for 12 h. The number of viable colonies on the plates was manually counted to evaluate the bactericidal effect.

To further assess clinical relevance, the antibacterial efficacy of Textilinin-1 was evaluated against a clinical *S. aureus* isolate derived from a diabetic foot ulcer. Textilinin-1 was dissolved to a stock concentration of 24.48 mg/mL. The assay was conducted in a 96-well plate, where 100 µL of the test agent, positive controls (Penicillin at 50 µg/mL or Ciprofloxacin at 10 µg/mL), or vehicle control (PBS) were mixed with 100 µL of 2× concentrated culture medium to prevent nutrient dilution. Subsequently, 10 µL of the clinical bacterial suspension was inoculated into each well. The final concentration of Textilinin-1 in the assay was 12.24 mg/mL. The plates were incubated at 37 °C with shaking at 125 rpm, and bacterial growth was monitored by measuring the optical density at 595 nm (OD_595_) every 6 h for a total duration of 36 h. Representative plates from each group were photographed under identical lighting conditions using a high-resolution digital camera. All experiments were conducted in three independent biological replicates.

The concentrations of Textilinin-1 differed across assays according to assay purpose and experimental context. Concentrations of 2–8 μM were used in mammalian wound-related cell assays to evaluate direct cellular responses, whereas 16 μM was used for topical application in the in vivo wound models to maintain local exposure at the wound surface. For the clinical *S. aureus* isolate assay, 12.24 mg/mL Textilinin-1 was used as an exploratory high-concentration condition to assess whether antibacterial activity could be detected against a clinically derived wound isolate. This concentration should not be interpreted as an optimized therapeutic concentration.

### 2.6. Cell Proliferation Assay

Proliferation of NIH/3T3 and SVEC4-10 cells was quantified using the Cell Counting Kit-8 (CCK-8; Abcam, Waltham, MA, USA) [29,32]. Cells were seeded at a density of 5 × 10^3^ cells per well into 96-well plates containing 100 μL of complete growth medium and incubated overnight to allow for cell attachment. The medium was then replaced with fresh medium containing Textilinin-1 at final concentrations of 0, 2, 4, or 8 μM. At predetermined time points (12, 24, and 48 h), CCK-8 reagent was added directly to each well, followed by incubation at 37 °C for 1 h. Absorbance at 450 nm was measured using a microplate reader (Infinite 200 PRO, TECAN, Switzerland).

Proliferation of human primary keratinocytes and normal NFF was evaluated using the trypan blue exclusion method. A 0.4% trypan blue stock solution was diluted 1:10 in 0.9% NaCl to obtain a final concentration of 0.04% [33]. Immediately after thawing, cell suspensions were mixed with the diluted trypan blue solution at a 1:5 ratio, and viable cells were counted microscopically using a Neubauer counting chamber. Cell viability was assessed in quintuplicate, with at least 500 cells counted per replicate, and expressed as the mean percentage of viable cells. For keratinocyte proliferation assays, human primary keratinocytes at passage 4 were seeded into 24-well plates at a density of 3 × 10^4^ cells per well in keratinocyte growth medium. After 48 h of culture, cells were treated with aprotinin or Textilinin-1 (2, 4, or 8 μM) and maintained for an additional five days before harvesting and counting. For fibroblast proliferation assays, NFF cells at passage 8 were seeded into 24-well plates at a density of 5 × 10^4^ cells per well. Following a 48 h attachment period, cells were treated with aprotinin or Textilinin-1 at the indicated concentrations and cultured for a further six days. At the designated endpoints, cells were harvested, stained, and enumerated as described above.

### 2.7. Wound Healing Assay in Vitro

NIH/3T3 cells were seeded into 6-well plates at a density of 2 × 10^5^ cells per well and incubated until confluence was reached. After 24 h, a linear scratch was created across the cell monolayer using a sterile 200 μL pipette tip [29]. Detached cells and debris were removed by washing twice with PBS. Cells were then cultured in serum-free medium containing Textilinin-1 at final concentrations of 0, 2, 4, or 8 μM. Migration into the scratched area was observed and photographed at 0, 12, 24, and 48 h post-scratch using phase-contrast microscopy (4× objective). Wound closure was quantified using ImageJ software (version 1.53q).

Human primary keratinocytes, isolated from neonatal foreskin tissue at passage 4 (P4), were seeded into 24-well plates at a density of 5 × 10^4^ cells per well in keratinocyte growth medium. Upon reaching confluence, cells were cultured in basal keratinocyte medium for an additional two days before wounding. A linear scratch was made using a sterile 200 μL pipette tip, and detached cells were removed with PBS. Cells were treated with EGF (20 ng/mL), aprotinin (4 μM), or Textilinin-1 (2, 4, or 8 μM). Each experimental condition was tested in quadruplicate. Real-time migration into the wound area was continuously recorded for 24 h using a videomicroscopy system equipped with a temperature- and CO_2_-controlled incubation chamber (37 °C, 5% CO_2_), with images captured at 30 min intervals. Human primary dermal fibroblasts at passage 8 (P8) were seeded into 24-well plates at a density of 6 × 10^4^ cells per well in DMEM supplemented with 10% fetal calf serum (FCS). After reaching confluence, cells were maintained in serum-free DMEM for two days, followed by wounding with a sterile 200 μL pipette tip. Detached cells were removed by gentle PBS washing. Cells were then treated with EGF (2 ng/mL), aprotinin (4 μM), or Textilinin-1 (2, 4, or 8 μM). Each treatment was performed in quadruplicate. Migration was monitored under standard culture conditions using a videomicroscopy system, with images acquired by time lapse camera every 15 min for 24 h.

The percentages of wound closure at 0, 12, and 24 h post-scratch were calculated using the formula: migration area (%) = (A_0_ − A_n_)/A_0_ × 100, where A_0_ represents the initial wound area, and A_n_ represents the residual wound area measured at each indicated time point.

### 2.8. Transwell Assay

In NIH/3T3 and SVEC4-10 cells, Transwell migration assays were performed using 24-well plates equipped with Transwell inserts containing polycarbonate membranes with an 8.0 μm pore size (Merck, MA, USA) [34]. Cells were seeded into the upper chambers at a density of 1 × 10^4^ cells per well. Cells in the upper chambers were treated with Textilinin-1 at final concentrations of 0, 2, 4, or 8 μM, while the lower chambers were filled with 600 μL of complete growth medium. Following incubation at 37 °C in a humidified atmosphere containing 5% CO_2_ for 12, 24, or 48 h, non-migrated cells remaining on the upper membrane surface were gently removed using a cotton swab. Cells that had migrated to the lower membrane surface were fixed with 4% paraformaldehyde (PFA) for 15 min and stained with 2% crystal violet for 6 min.-Stained cells were visualized using a light microscope (20× objective), and migration was quantified by counting cells in five randomly selected microscopic fields per well.

### 2.9. ROS Eliminate Assay

NIH/3T3 cells were seeded into 24-well plates at a density of 1 × 10^5^ cells per well. Intracellular reactive oxygen species (ROS) levels were assessed using the fluorescent probe 2′,7′-dichlorofluorescein diacetate (DCFH-DA; Sigma-Aldrich, St. Louis, MO, USA) [35]. Cells were treated with 100 μM hydrogen peroxide (H_2_O_2_) in the presence or absence of Textilinin-1 at final concentrations of 0, 2, 4, or 8 μM and incubated for 24 h at 37 °C under standard humidified conditions (5% CO_2_). Following treatment, cells were loaded with DCFH-DA in accordance with the manufacturer’s instructions to detect intracellular ROS production. Fluorescence images were acquired using a confocal fluorescence microscope (Leica SP8, Wetzlar, Germany) equipped with a 10× objective lens. ROS accumulation was quantified by measuring the mean fluorescence intensity using ImageJ software (version 1.54g; National Institutes of Health, Bethesda, MD, USA).

### 2.10. Tube Formation Assay

EHS matrix extract (Merck, MA, USA) was diluted 1:1 with serum-free medium, dispensed into 96-well plates, and incubated at 37 °C for 1 h to allow polymerization and gel formation. After solidification, SVEC4-10 cells suspended in low-serum medium (1% FBS) were seeded onto the polymerized matrix at a density of 2 × 10^4^ cells per well. Cells were incubated at 37 °C for 4 h under standard culture conditions, after which tube formation was evaluated using a light microscope equipped with a 10× objective lens. Angiogenic activity was quantified by counting the number of nodes formed in each well [35].

### 2.11. Cell Adhesion Assay

For human primary keratinocytes, an adhesion assay was performed under non-coated conditions to evaluate the effect of Textilinin-1 on cell adhesion. Human primary keratinocytes, isolated from neonatal foreskin, were used at passage 5 (P5). Cells were seeded into 24-well plates without collagen coating at a density of 3 × 10^4^ cells per well in keratinocyte growth medium. At 24 h post-seeding (Day 1), the medium was replaced with fresh keratinocyte growth medium containing Textilinin-1 at the specified concentrations or vehicle control. Cell adhesion was assessed on Days 2 and 14 by inverted phase-contrast microscopy. At each time point, representative fields were imaged to qualitatively evaluate cell attachment and spreading. All experimental conditions were conducted in triplicate wells.

### 2.12. RNA Extraction and qPCR

Cells were harvested at designated time points, and total RNA was extracted using TRIzol reagent (Thermo Fisher Scientific, Waltham, MA, USA) according to the manufacturer’s instructions. For each sample, 500 ng of total RNA was reverse transcribed into complementary DNA (cDNA) using the PrimeScript RT Reagent Kit (Takara, Bio Inc., Kusatsu, Shiga, Japan). Quantitative PCR (qPCR) was performed with SYBR Green Master Mix (Applied Biosystems, Waltham, MA, USA) on a QuantStudio™ 5 Real-Time PCR System (Thermo Fisher Scientific) equipped with fluorescence detection. Gene expression was analyzed in different cell types and mouse skin tissues. Specifically, in NIH/3T3 fibroblasts, target genes included *CyclinD1*, *Cdk2*, *Pdgf*, *Bcl-2*, *Atg13*, *Erk1*, *and Erk2*; *in* SVEC4-10 endothelial cells, *CyclinD1*, *Cdk2*, *Pdgf*, *Bcl-2*, *Atg13*, *Erk1*, *Erk2*, *Vegfc*, *Vegfd*, *Vegfr2*, *Ccl2*, *Cxcl1*, and *Cxcl10*; and in RAW 264.7 macrophages, *Arg1, Cxcl1, Cxcl10, Tnf-α,* and *iNos*. In mouse skin tissue samples, expression of *Tnf, Il-1β, Tgfβ, Vegf, Col1a1, Col3a1*, and *Pdgf* was assessed [35]. Primer sequences for all qPCR assays are provided in Table S1. Relative gene expression was calculated using the 2^−ΔΔCt^ method, with *Actb* serving as the internal reference for normalization.

### 2.13. Wound Healing Assessment in Mouse Wound Model

The wound healing efficacy of Textilinin-1 was evaluated in vivo using a full-thickness excisional wound model in male C57BL/6J mice (aged 8–10 weeks and weighing 20–25 g) [36]. All animals were housed within the University of Queensland Biological Resources animal facility in a pathogen-free environment with 12 h/12 h dark/light cycle and free access to food and water. Mice were randomly assigned to either the control group (PBS) or the Textilinin-1-treated group (16 μM in PBS), with ≥6 mice per group. All procedures for the mice work were approved by the Animal Ethics Committee of the University of Queensland (Approval No: 2022/AE000346, approved on 1 January 2023) and conducted in compliance with institutional guidelines. Mice were anesthetized with inhaled isoflurane, and the dorsal surface was shaved with an electric shaver, followed by sterilization using povidone–iodine and 80% ethanol. A circular full-thickness excisional wound (~5 mm diameter) was created on the dorsum of each mouse using a sterile biopsy punch and surgical scissors. Animals were housed individually with ad libitum access to food and water. Digital images of wounds were captured on days 0, 3, 7, and 14 post-injury, and wound areas were quantified using ImageJ software. At each time point, wound tissues from at least six randomly selected mice per group were harvested post-euthanasia (carbon dioxide asphyxiation) for subsequent analyses. The wound healing rate was calculated using the formula:

Wound healing percentage (%) = [(wound area at baseline—wound area at the indicated time point)/wound area at baseline] × 100

### 2.14. Wound Healing Assessment in Pig Wound Model

A porcine excisional wound model was established in one healthy adult pig (30–40 kg). All procedures for the procine work were approved by the Animal Ethics Committee of the University of Queensland (Approval No: CICR/196/06/CICR, approved on 2006) January and conducted in compliance with institutional guidelines After general anesthesia and aseptic preparation of the dorsal area, eight full-thickness square wounds (≈6 cm diameter) were created at day 0, four on each side of the midline, extending through the dermis to the panniculus carnosus. Wounds on the left side received topical Textilinin-1 (16 μM), while those on the right side were treated with saline as controls. Treatments were applied immediately after wound creation and reapplied once every two or three days. Following each application, wounds were covered with a sterile, breathable dressing to maintain drug contact and protect the wound bed.

Standardized digital photographs were taken at fixed distance with a calibration scale at designated time points (e.g., days 0, 3, 5, 7, 10, 12, 14, and 17). Wound area was quantified in ImageJ using the calibration scale, and wound healing percentage at each time point was calculated as (A0 −At)/A0 × 100, where *A*0 is the baseline area (day 0) and *At* is the area at time *t*. Healing rate (mm^2^/day) was derived from serial area changes between consecutive time points. Image analysis was performed by investigators blinded to group allocation. Re-epithelialization was quantified from representative H&E-stained sections of day 0, 3, 5, 7, 10, 12, 14, 17 at 2× magnification. Measurements were performed on digitized images by blinded assessors.

### 2.15. Histological Analysis

At postoperative days 7 and 14, mice were euthanized via carbon dioxide asphyxiation, and wound skin tissues were collected for histological analysis. Excised samples were fixed in 4% PFA at 4 °C overnight, followed by graded ethanol dehydration. Tissues were then embedded in paraffin and sectioned at a thickness of 5 μm. Paraffin sections were stained with hematoxylin and eosin (H&E) staining for general histopathological evaluation and with Masson’s trichrome (MT) staining to assess collagen deposition [36].

### 2.16. Protein-Protein Docking

To explore potential interactions between Textilinin-1 and selected protein targets, protein–protein docking was conducted using the HDOCK server (http://hdock.phys.hust.edu.cn/) (accessed on 4 February 2025). The three-dimensional structures of Textilinin-1 and all target proteins were obtained from the Protein Data Bank (PDB) in PDB format. Protein and ligand structures were uploaded to the server, and docking was performed in protein–small molecule mode with default parameters. For each target, HDOCK generated the top 10 docking models ranked by docking score, and the highest-scoring model (Top 1) was selected for further analysis.

### 2.17. Statistical Analysis

All data are expressed as the mean ± standard deviation (SD) from at least three independent biological replicates, unless otherwise stated. Statistical analyses were performed using GraphPad Prism software (version 9.0; GraphPad Software, San Diego, CA, USA). When the two groups do not have homogeneity of variance and conform to normal distribution, Student’s *t* test is used. One-way analysis of variance (ANOVA) followed by Tukey’s multiple comparisons test was used to compare the different parameters between the groups at baseline, with a *p* value < 0.05 considered significant. Tukey’s post hoc tests were performed only if F-statistic achieves statistical significance (*p* < 0.05) and that there is no significant heteroscedasticity. Levels of significance are denoted as follows: *p* < 0.05 (*), *p* < 0.01 (**), and *p* < 0.001 (***). For mouse wound healing measurements over time, wound areas were assessed longitudinally in the same mice at the indicated time points. Group comparisons between control and Textilinin-1-treated mice were performed at each individual time point using unpaired two-tailed Student’s t-tests. For the pilot porcine study, data were treated as wound-level observations from a single animal, comprising four Textilinin-1-treated wounds and four control wounds. These porcine data were presented descriptively and were not considered as independent biological replicates for definitive statistical inference. No animals, wounds, or data points were excluded from the analysis.

## 3. Results

### 3.1. Bioinformatic Prediction of Textilinin-1 Targets and Functional Implications in Wound Healing

**Figure 1:**
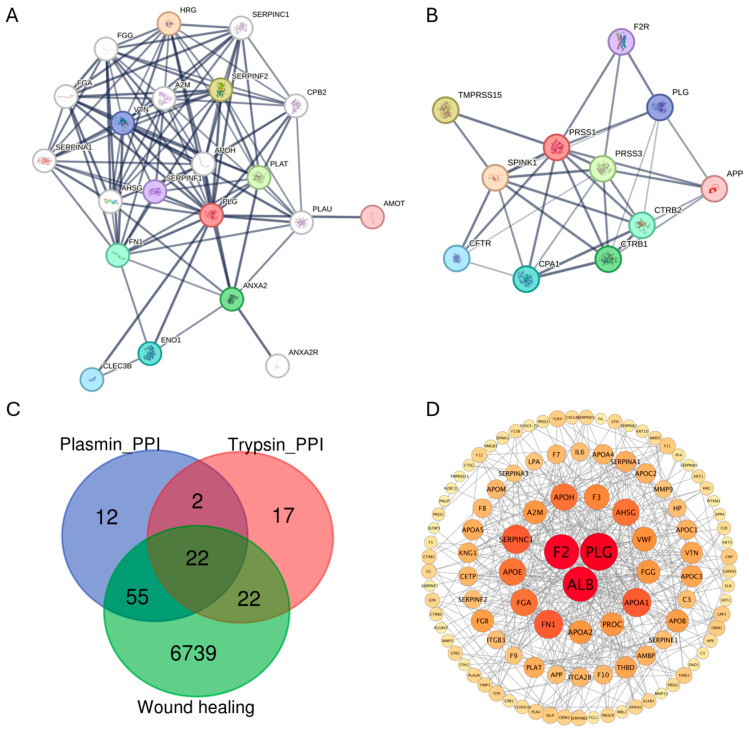
Bioinformatics analysis of potential targets related to Textilinin-1 in wound healing. (**A**,**B**) Protein–protein interaction (PPI) networks for Plasmin (PLG) (**A**) and Trypsin (PRSS1) (**B**) were constructed using the STRING database. (**C**) Venn diagram showing the overlap among Plasmin PPI-associated genes, Trypsin PPI-associated genes, and genes related to wound healing. (**D**) Network visualization of intersection genes identified in panel (**C**), highlighting core targets.

To elucidate the potential molecular mechanisms by which Textilinin-1 contributes to wound healing, we first performed target prediction and network analysis. Textilinin-1 is a Kunitz-type serine protease inhibitor with two experimentally validated targets, PLG and PRSS1. PPI networks cantered on PLG and PRSS1, revealed 130 interacting proteins that may represent potential downstream effectors in the Textilinin-1 signalling context (Figure 1A,B). In parallel, a GeneCards search retrieved 6838 wound healing-associated genes. Venn diagram analysis revealed 99 genes that were present in both PRSS1- and PLG-derived PPI networks and annotated as wound healing associated (Figure 1C). PPI mapping of these overlapping genes highlighted a dense interaction network in the wound healing context (Figure 1D), suggesting a coordinated role in tissue repair and implicating Textilinin-1 as a potential regulator of wound healing.

**Figure 2:**
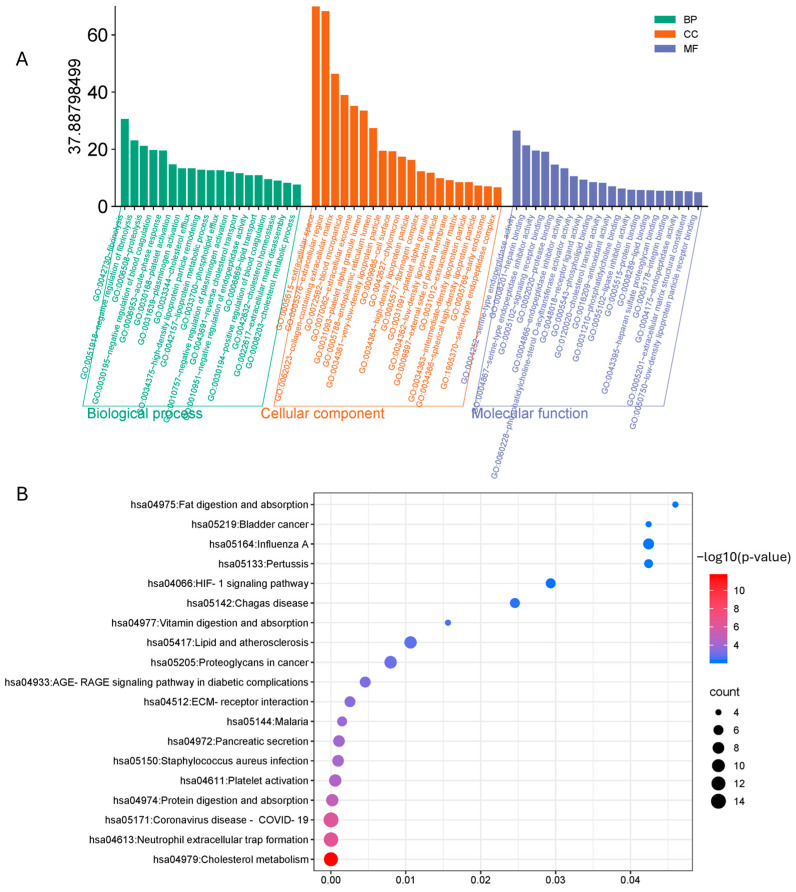
Bioinformatics analysis of potential pathways related to Textilinin-1 in wound healing. (**A**) Gene ontology (GO) enrichment analysis of intersection genes, categorized into biological process (BP), cellular component (CC), and molecular function (MF). (**B**) Kyoto Encyclopedia of Genes and Genomes (KEGG) pathway enrichment analysis of intersection genes, with dot size representing gene count and color indicating statistical significance (−log_10_(*p*-value)).

To further characterize the biological roles of these overlapping genes, GO enrichment and KEGG pathway enrichment analyses were conducted. GO enrichment analysis of the 99 genes showed significant enrichment (*p* < 0.05) across biological processes (BP), cellular components (CC), and molecular functions (MF) (Figure 2A). The top BP terms included blood coagulation, fibrinolysis, plasminogen activation, and regulation of proteolysis. CC enrichment was observed in the extracellular space, collagen-containing extracellular matrix, blood microparticles, extracellular exosomes, and platelet alpha granule lumen. MF enrichment was dominated by serine-type endopeptidase activity, receptor ligand activity, calcium ion binding, and extracellular matrix structural constituents. KEGG pathway analysis (DAVID) identified several significantly enriched pathways, including complement and coagulation cascades, pancreatic secretion, Staphylococcus aureus infection, cholesterol metabolism, and the AGE–RAGE signalling pathway in diabetic complications (Figure 2B), highlighting links between Textilinin-1-associated networks and inflammatory, thrombotic, and metabolic aspects of wound healing.

### 3.2. Textilinin-1 Promotes the Function of Key Cells in Wound Healing

To determine the functional effects of Textilinin-1 in skin repair, we assessed its impact on the proliferation, migration, and adhesion of fibroblasts, endothelial cells, and keratinocytes, which are the primary cell types involved in skin repair.

#### 3.2.1. Textilinin-1 Promotes Proliferation and Migration of Mouse Endothelial Cells (SVEC4-10) and Fibroblasts (NIH/3T3)

**Figure 3:**
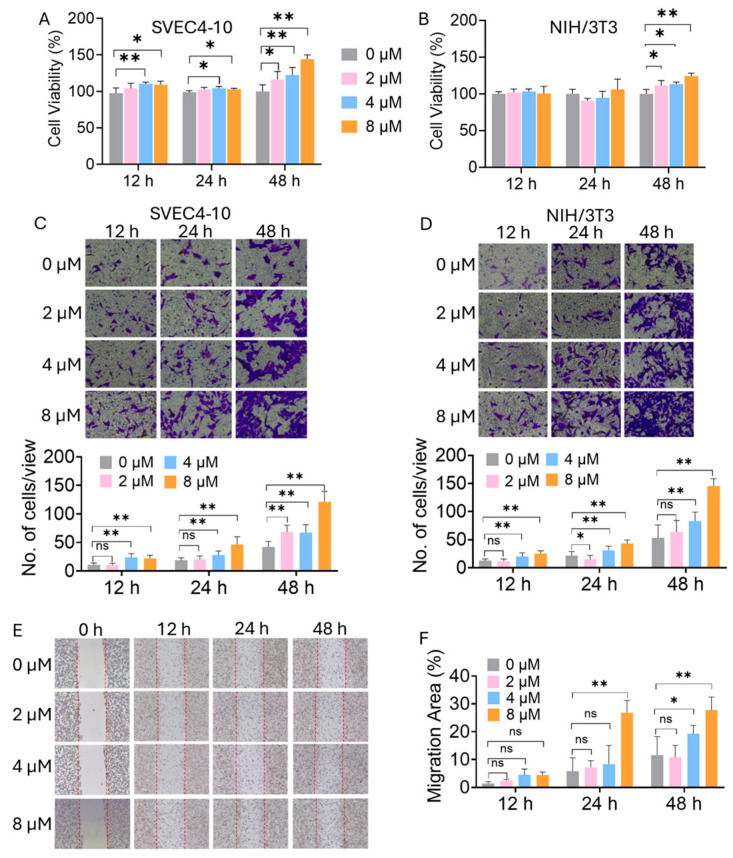
Textilinin-1 stimulates proliferation, migration of mouse endothelial cells (SVEC4-10) and fibroblasts (NIH/3T3). (**A**,**B**) Cell viability after Textilinin-1 treatment (0, 2, 4, 8 μM) for 12, 24, or 48 h measured by CCK-8 (*n* = 5). (**C**,**D**) Transwell migration of SVEC4-10 and NIH/3T3 after Textilinin-1 treatment (0, 2, 4, 8 μM) for 12, 24, or 48 h, shown as representative images (20×) and quantification (*n* = 3). (**E**,**F**) NIH/3T3 wound healing assay after Textilinin-1 treatment (0, 2, 4, 8 μM) at 12, 24, or 48 h, shown as representative images (4×) and overall quantification (*n* = 3). Data are presented as mean ± SD. * *p* < 0.05, ** *p* < 0.01, ns: not significant.

In SVEC4-10 endothelial cells, cell viability assessed by the CCK-8 assay increased significantly following 48 h treatment with 2–8 μM Textilinin-1, with the highest proliferation rate observed at 8 μM (*p* < 0.01; Figure 3A). Transwell assays confirmed a substantial increase in the number of migrated cells, particularly at 8 μM (*p* < 0.01; Figure 3C). Consistent effects were observed in NIH/3T3 fibroblasts. CCK-8 analysis revealed significantly increased viability after 48 h treatment with 8 μM Textilinin-1 (*p* < 0.01; Figure 3B), while lower concentrations produced moderate but significant increases. Transwell migration assays demonstrated a clear concentration-dependent increase in fibroblast motility, with the highest number of migrated cells observed in the 8 μM group at 12 h, 24 h and 48 h (*p* < 0.01; Figure 3D). Wound healing assays similarly showed that Textilinin-1 markedly accelerated wound closure at both 24 h and 48 h in a concentration-dependent manner (*p* < 0.01; Figure 3E,F). Together, these results indicate that the presence of Textilinin-1 robustly stimulates proliferation and migration in both endothelial and fibroblast populations, highlighting its pro-regenerative potential during wound healing.

#### 3.2.2. Textilinin-1 Promotes Proliferation and Migration of Human Primary Fibroblasts

**Figure 4:**
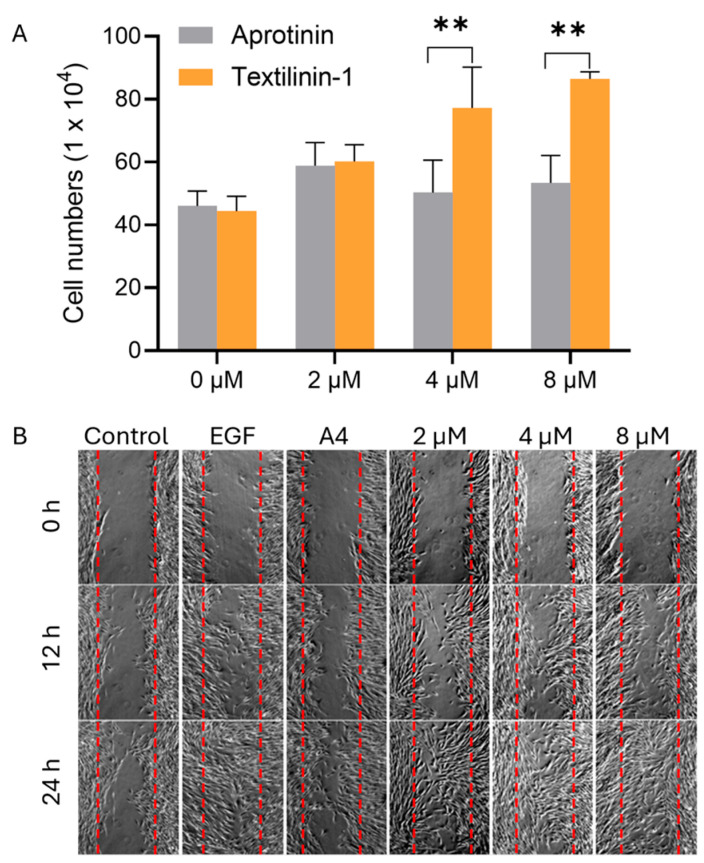
Textilinin-1 treatment promotes proliferation and migration of human primary fibroblasts. (**A**) Quantification of NFF cell numbers following treatment with Aprotinin or Textilinin-1 at the indicated concentrations for 6 days, as assessed by trypan blue exclusion staining (*n* = 4). (**B**) Representative images of wound healing assays at 0, 12, and 24 h after treatment with control, EGF(2 ng/mL), A4(4 μM), or various concentrations of Textilinin-1 (2, 4, and 8 μM) (*n* = 4). Data are presented as mean ± SD. ** *p* < 0.01.

Textilinin-1 treatment enhanced the regenerative activity of human primary fibroblasts, promoting proliferation, migration, and cell viability in a concentration-dependent manner, with maximal efficacy observed at 8 μM (*p* < 0.01; Figure 4A) This proliferative effect exceeded that of Aprotinin at equivalent concentrations, and no cytotoxicity was observed across the tested range. Wound healing assays showed increased fibroblast migration following Textilinin-1 treatment. Compared with control and Aprotinin-treated groups, wound closure was significantly faster at 4 μM and 8 μM, with visible narrowing of the wound gap at 12 h and 24 h (Figure 4B). Together, these results demonstrate that Textilinin-1 treatment is associated with increased proliferation and migration of human primary fibroblasts, supporting its strong pro-regenerative potential in vitro.

#### 3.2.3. Textilinin-1 Promotes Adhesion-, Proliferation-, and Migration-Related Responses of Human Keratinocytes

**Figure 5:**
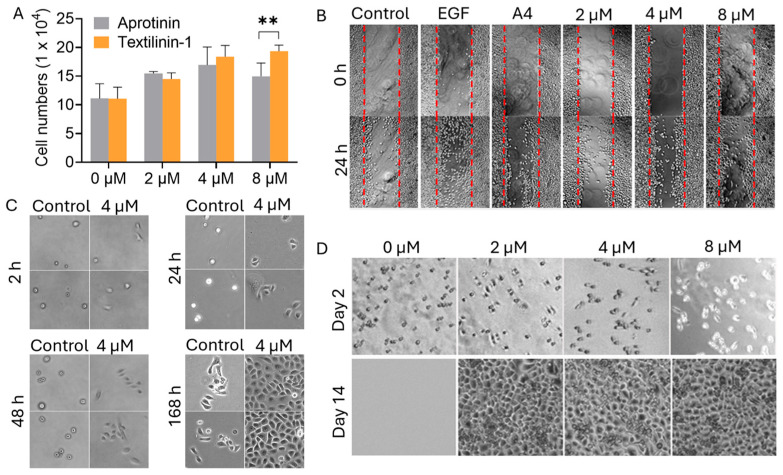
Textilinin-1 enhances adhesion, proliferation and migration of human primary keratinocytes. (**A**) Quantification of human primary keratinocyte numbers following treatment with aprotinin or Textilinin-1 at the indicated concentrations for 5 days (*n* = 4). (**B**) Representative images of wound healing assays at 0 and 24 h after treatment with control, EGF (20 ng/mL), A4 (Aprotinin, 4 μM), or various concentrations of Textilinin-1 (2, 4, and 8 μM) (*n* = 4). (**C**,**D**) Representative phase-contrast microscopy images showing cell adhesion and spreading on Day 2 and Day 14 after treatment with 0, 2, 4, or 8 μM Textilinin-1 (*n* = 4). Data are presented as mean ± SD., ** *p* < 0.01.

To evaluate whether and how Textilinin-1 effects on keratinocyte proliferation and migration, primary human keratinocytes were then treated with varying concentrations of Textilinin-1 (2, 4, and 8 μM), with Aprotinin serving as a reference compound. As shown in Figure 5A, Textilinin-1 treatment promoted keratinocyte proliferation in a concentration-dependent manner. Treatment with 2–8 μM Textilinin-1 significantly increased cell numbers, with the increase observed at 8 μM. This pro-proliferative activity exceeded that of Aprotinin at equivalent concentrations. In the wound-healing assay, Textilinin-1 treatment was associated with increased wound closure compared with the control and aprotinin-treated groups. At 24 h, 8 μM treatment group achieved a degree of wound closure comparable to that of the EGF-treated positive control (Figure 5B). The effects of Textilinin-1 on keratinocyte adhesion and spreading were first examined at a single concentration (4 μM) across different time points (Figure 5C). Within 2h of seeding, a greater proportion of cells in the Textilinin-1 group had adhered to the substrate and extended protrusions compared with the untreated control. By 24 h and 48 h, cell spreading was more extensive, and intercellular connections began to form. After 7 days, Textilinin-1 treated cells formed an almost continuous monolayer with tight cell–cell junctions and nearly covered the plate surface completely (Figure 5D). We then assessed the effects across concentrations after 14 days, all Textilinin-1 treated cells showed improved monolayer formation, with maximal confluence and cell density achieved at 8 μM. Collectively, these findings demonstrate that Textilinin-1 treatment is associated with increased keratinocyte adhesion, proliferation, and migration.

### 3.3. Textilinin-1 Promotes Cutaneous Wound Healing In Vivo

To evaluate whether the regenerative effects of Textilinin-1 observed in vitro translate into in vivo wound healing, full-thickness wound models were established in mice and a preliminary pilot study was conducted in a porcine model.

**Figure 6:**
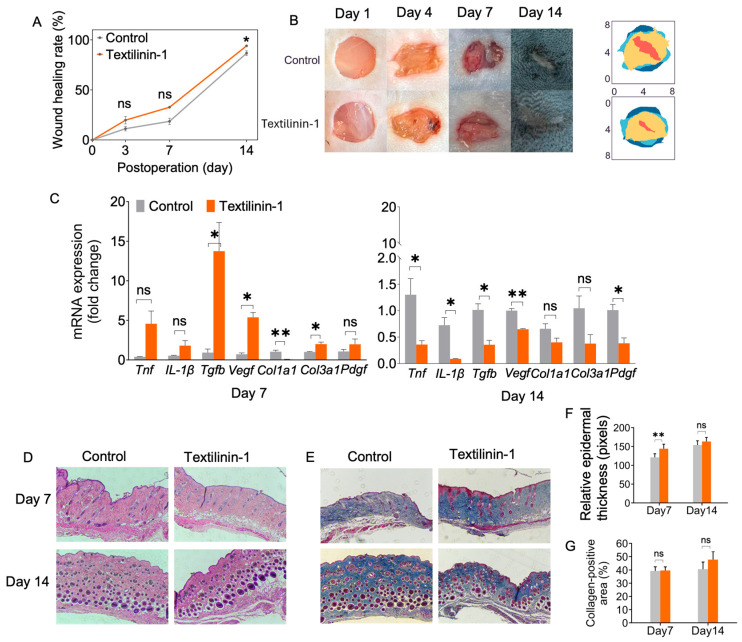
Textilinin-1 accelerates wound healing and modulates gene expression in a mice excisional wound model. (**A**) Wound healing rate over time in control and Textilinin-1-treated groups (*n* = 6). (**B**) Representative wound images and wound area measurements showing wound appearance at Day 1, 4, 7, and Day 14 post-injury (*n* = 6). (**C**) qPCR analysis of wound tissue showing relative mRNA expression of *Tnf*, *IL-1β*, *Tgfb*, *Vegf*, *Col1a1*, *Col3a1*, and *Pdgf* on Day 7 and Day 14, as determined by qPCR (*n* = 6). (**D**,**E**) Representative H&E (4×) and Masson’s trichrome (4×) staining of wound tissue sections from control and Textilinin-1-treated mice at Day 7 and Day 14. (**F**) Image-based quantification of relative epidermal thickness from H&E-stained sections, epidermal thickness was measured as a relative pixel-based value. (**G**) Quantification of collagen-positive area from Masson’s trichrome-stained sections. Data are presented as mean ± SD. * *p* < 0.05, ** *p* < 0.01, ns: not significant.

Based on in vitro findings, 16 μM Textilinin-1 was applied topically to full-thickness skin wounds in mice, while control group mice received PBS alone. Topical Textilinin-1 treatment accelerated wound closure compared with controls, as reflected by a more rapid reduction in wound area over time (Figure 6A,B). This pro-healing effect became increasingly evident as healing progressed, with the treated group maintaining a consistently greater degree of closure by day 14 post-injury, indicating sustained enhancement of tissue repair (*p* < 0.05; Figure 6A,B). To explore the translational potential of these findings in skin that more closely resembles human anatomy, we further evaluated Textilinin-1 in a pilot porcine full-thickness wound model. In this proof-of-concept assessment, Textilinin-1 treatment not only significantly shortened the time required for re-epithelialization and wound closure but also reduced visible bleeding at the wound site (Figure S2). mRNA expression of key inflammatory and regenerative genes was analysed in the mouse wound tissues. Expression of *IL-1β* and *Tnf* showed an increase trend in the Textilinin-1 group relative to controls at day 7 (*p* > 0.05, Figure 6C). By day 14, expression of both cytokines was significantly reduced in Textilinin-1-treated wounds compared with controls (*p* < 0.05; Figure 6C).

Textilinin-1 upregulated the expression of canonical wound-healing genes. We next examined the expression of canonical wound-healing genes, including *Tgfb, Vegf, Col1a1, Col3a1,* and *Pdgf.* Textilinin-1 treatment upregulated *Tgfb, Vegf,* and *Col3a1* compared with controls, consistent with a pro-regenerative transcriptional profile during the early phase of healing at day 7 (*p* < 0.05, *p* < 0.05, and *p* < 0.01, respectively). Interestingly, *Col1a1* expression was lower in the Textilinin-1 group at this time point (*p* < 0.05; Figure 6C). By day 14, the mRNA levels of *Tgfb, Vegf,* and *Pdgf* were reduced in Textilinin-1-treated wounds, and both *Col1a1* and *Col3a1* displayed a downward trend, although these latter changes were not statistically significant (Figure 6C).

Histological analysis further supported enhanced tissue regeneration in Textilinin-1-treated wounds. H&E staining showed that Textilinin-1-treated wounds exhibited relatively hypocellular granulation tissue, and a thin, immature epithelium compared with controls, yet epidermal and subepidermal structures appeared more organized and structurally intact at day 7 (Figure 6D). By day 14, Textilinin-1 treatment resulted in enhanced granulation tissue maturation, more complete re-epithelialization, and improved dermal remodelling relative to untreated wounds, indicating a positive effect on tissue regeneration (Figure 6D). Image-based quantification showed that relative epidermal thickness was increased in Textilinin-1-treated wounds at Day 7 compared with controls (*p* < 0.01; Figure 6F), whereas no significant difference was observed at Day 14 (Figure 6F).

MT staining revealed markedly greater granulation tissue formation and collagen deposition (blue staining) in Textilinin-1-treated wounds compared with controls at day 7 (Figure 6E). In the treated group, the basal lamina and keratin layer were well organized and clearly delineated, whereas control wounds showed loosely arranged granulation tissue with reduced collagen content. The dermal layer in controls displayed higher cellularity and a reticular architecture, with the basal lamina not yet fully formed (Figure 6E). These histological differences persisted through day 14. Quantitative image analysis showed a higher collagen-positive area in Textilinin-1-treated wounds at Day 14, although this difference did not reach statistical significance (Figure 6G). At Day 7, collagen-positive area was similar between groups (Figure 6G).

Together, these results demonstrate that Textilinin-1 treatment is associated with accelerated cutaneous wound closure, dynamic inflammatory and repair-related gene expression, increased relative epidermal thickness at Day 7, and changes in collagen deposition during tissue repair.

### 3.4. Mechanistic Insights into Textilinin-1-Associated Wound Repair

To explore preliminary mechanistic hypotheses for future investigation of Textilinin-1, we evaluated how it influences regenerative gene expression, oxidative and angiogenic responses, macrophage activation, and bacterial growth.

#### 3.4.1. Textilinin-1 Upregulates Expression of Proliferation and Migration-Related Genes in SVEC4-10 and NIH/3T3 Cells

**Figure 7:**
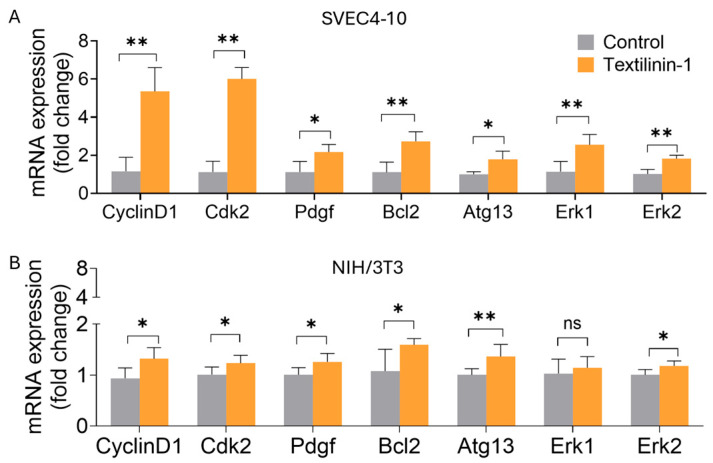
Textilinin-1 stimulates proliferation and migration-related gene expression in SVEC4-10 cells and NIH/3T3 cells. (**A**) Relative mRNA expression levels of genes associated with proliferation and migration in SVEC4-10 cells following Textilinin-1 treatment, as determined by qPCR (*n* = 6). (**B**) Relative mRNA expression levels of genes associated with proliferation and migration in NIH/3T3 cells following Textilinin-1 treatment, as determined by qPCR (*n* = 6). Data are presented as mean ± SD. * *p* < 0.05, ** *p* < 0.01, ns: not significant.

Our transcriptional analysis suggested that Textilinin-1 is associated with pro-regenerative cellular response, particularly in genes related to intracellular signalling cascades, rather than marked changes in the expression of extracellular matrix components. To dissect the molecular mechanisms associated with the observed regenerative phenotype, we analysed transcriptional profiles in SVEC4-10 endothelial cells and NIH/3T3 fibroblasts following 24 h treatment with 8 μM Textilinin-1. Quantitative PCR revealed statistically significant, although in some cases modest, increases in genes related to cell cycle progression and survival. In Figure 7A, Textilinin-1 treatment increased the expression of *Cyclin D1, Cdk2, Bcl2,* and *Erk1/2* (*p* < 0.01), accompanied by elevated *Pdgf* and *Atg13* levels (*p* < 0.05) in SVEC4-10 endothelial cells. NIH/3T3 fibroblasts displayed a complementary profile, characterized by a highly significant upregulation of the autophagy regulator *Atg13* (*p* < 0.01) and significant increases in *Cyclin D1, Cdk2, Pdgf, Bcl2,* and *Erk2* (*p* < 0.05, Figure 7B). In contrast, the machinery for matrix deposition and remodelling remained quiescent; no significant changes were detected in TGF-β signalling mediators (*Smad2/3/4*), myofibroblast markers (*α-SMA*), or matrix metalloproteinases (*Mmp2/9, Timp1/2, Col3a1*) (Figure S1). Collectively, these data suggest that Textilinin-1 enhances wound repair by intracellularly priming effector cells for division and survival, rather than by directly stimulating extracellular matrix synthesis.

#### 3.4.2. Antioxidant Response and Angiogenic Activation

**Figure 8:**
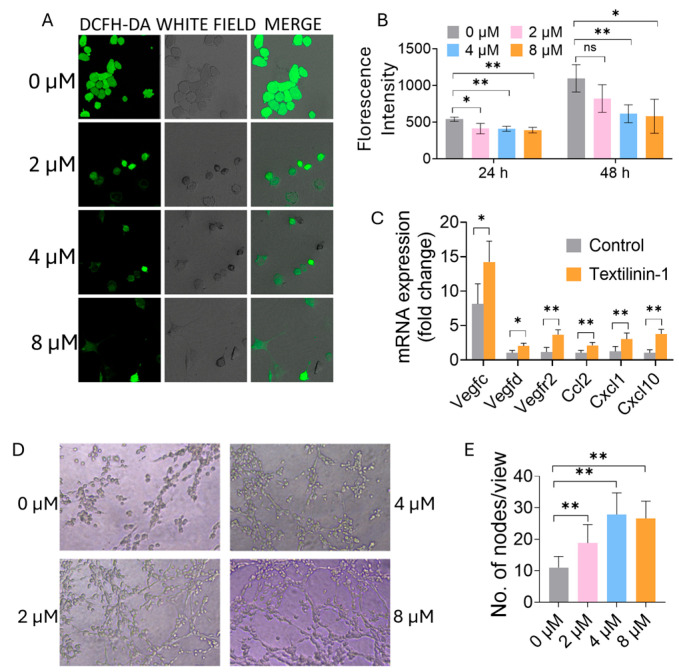
Anti-ROS production of fibroblasts and angiogenesis of endothelial cells. (**A**,**B**) Intracellular ROS levels in NIH/3T3 cells were attenuated after Textilinin-1 treatment (0, 2, 4, 8 μM), shown by a representative confocal image at 24 h (left) and quantified by fluorescence intensity at 24 and 48 h (*n* = 4). (**C**) Relative mRNA expression levels of genes related to angiogenesis in SVEC4-10 cells, determined by qPCR following Textilinin-1 treatment (*n* = 6). (**D**) Tube formation assays showing capillary-like structure formation (10×) after treatment with 0, 2, 4, or 8 μM Textilinin-1 (*n* = 4). (**E**) Quantification of the number of nodes per view in tube formation assays (*n* = 4). Data are presented as mean ± SD. * *p* < 0.05, ** *p* < 0.01, ns: not significant.

In NIH/3T3 fibroblasts, fluorescence microscopy images showed a concentration-dependent reduction in green fluorescence intensity, indicating attenuated ROS levels following treatment (Figure 8A). A high level of basal fluorescence was observed in the untreated control cells (0 µM), while a decrease was evident in cells treated with 4 µM and the lowest intensity was detected at 8 µM (Figure 8A). This observation was further quantified by mean fluorescence intensity, which confirmed that Textilinin-1 led to a significant and concentration-dependent decline in intracellular ROS levels, especially at 24 h (Figure 8B). These results collectively indicate that Textilinin-1 suppresses oxidative stress in the cells.

The angiogenic potential of Textilinin-1 was then assessed using a tube formation assay on SVEC4-10 cells. Untreated cells formed only rudimentary, poorly connected tubular structures (Figure 8D). In contrast, Textilinin-1 treated cells markedly enhanced vascular network complexity, with longer, more branched, and interconnected tubes emerging within 4 h in a concentration-dependent manner (Figure 8E). Notably, key pro-angiogenic factors, including *Vegfc, Vegfd, Vegfr2, Ccl2, Cxcl1,* and *Cxcl10*, were also strongly induced, suggesting activation of angiogenic signalling pathways (Figure 8C).

Collectively, the data suggest that Textilinin-1 treatment is associated with reduced ROS signal in fibroblasts and angiogenesis-related responses in endothelial cells under the tested conditions.

#### 3.4.3. Textilinin-1 Suppresses Pro-Inflammatory Gene Expression and M1-Like Inflammatory Activation in Macrophages

**Figure 9:**
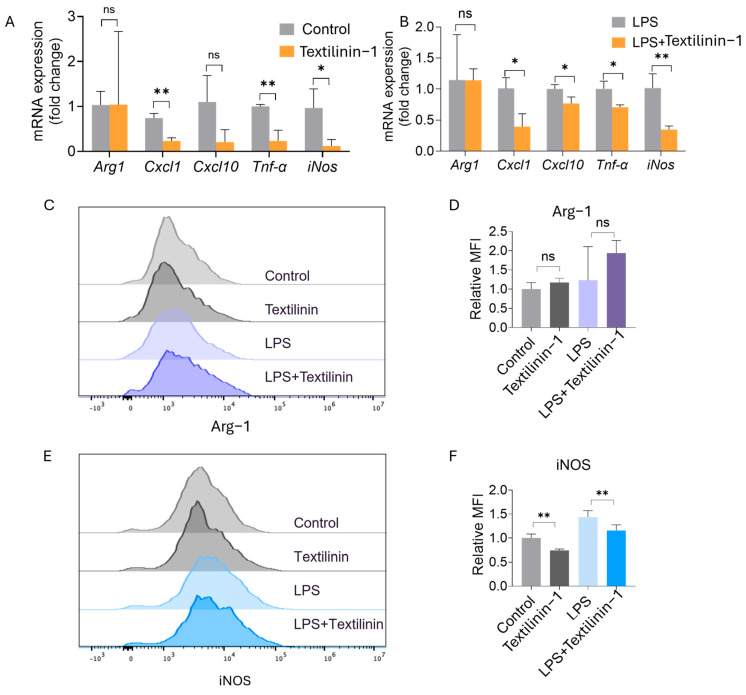
Textilinin-1 modulates macrophage M1-like inflammatory activation and inflammatory gene expression in vitro. (**A**,**B**) Relative mRNA expression levels of *Arg1, Cxcl1, Cxcl10, Tnf-α*, and *iNos* in RAW 264.7 macrophages following treatment with Textilinin-1, as determined by qPCR (*n* = 6). Panel A shows the effect of Textilinin-1 under basal conditions, with expression levels normalized to the untreated control group. Panel B shows the effect of Textilinin-1 under LPS-stimulated inflammatory conditions, with expression levels normalized to the LPS-treated group. (**C**–**F**) Flow cytometry analysis of Arg-1 and iNos protein expression in RAW 264.7 macrophages under different treatment conditions: control, 8 μM Textilinin-1, LPS, and LPS + 8 μM Textilinin-1 (*n* = 4). Representative histogram overlays (left) and quantification of mean fluorescence intensity (MFI, right) are shown. Data are presented as mean ± SD. * *p* < 0.05, ** *p* < 0.01, ns: not significant.

To assess whether Textilinin-1 affects inflammatory gene expression under both basal and inflammatory conditions, qPCR data were analysed separately in untreated and LPS-stimulated RAW 264.7 cells. qPCR analysis revealed that Textilinin-1 significantly reduced basal expression of key pro-inflammatory genes, including *Cxcl1, Tnf*, and *iNos* (Figure 9A). Notably, the M2 marker *Cxcl10* and *Arg1* expression remained unchanged, indicating selective regulation without induction of alternative activation. Upon LPS stimulation, Textilinin-1 potently attenuated the upregulation of *Cxcl1, Cxcl10, Tnf,* and *iNos* (Figure 9B), demonstrating a robust inhibitory effect on M1-like inflammatory activation. In contrast, *Arg1* expression was unaffected across all conditions, suggesting no promotion of M2 polarization at the transcriptional level (Figure 9A,B). Flow cytometric analysis confirmed these findings at the protein level. While Arg1 protein levels showed no significant variation across all treatments (Figure 9C,D), iNOS expression was markedly modulated (*p* < 0.01; Figure 9E). Textilinin-1 alone significantly reduced basal iNOS expression (from relative MFI ~1.6 to ~0.7), and co-treatment with LPS, Textilinin-1 significantly suppressed LPS-induced iNOS expression (changed to relative MFI ~1.3, *p* < 0.01) (Figure 9F). Collectively, these data demonstrate that Textilinin-1 selectively inhibits pro-inflammatory signalling and M1-like inflammatory activation in macrophages, without driving M2 phenotypic changes.

#### 3.4.4. Textilinin-1 Exhibits Antibacterial Activity Against *S. aureus* Under the Conditions Tested

**Figure 10:**
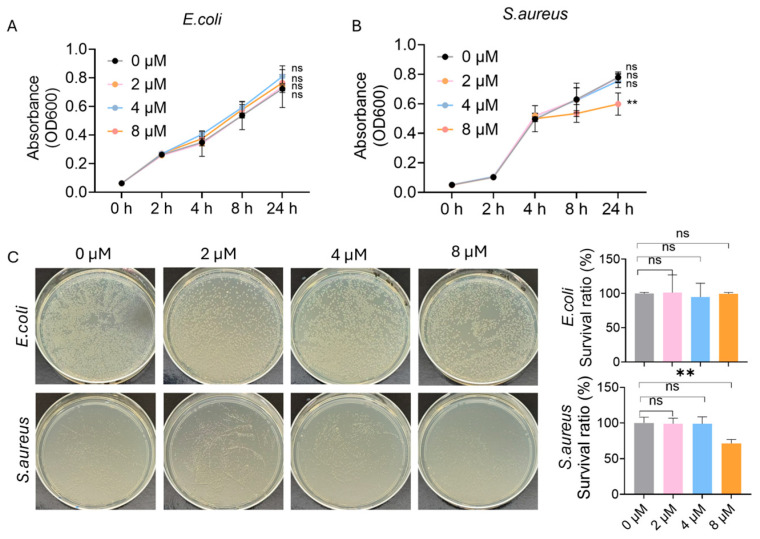
Antibacterial effects of Textilinin-1 against *E. coli* and *S. aureus* under the conditions tested. (**A**,**B**) Growth curve of *E. coli* and *S. aureus* treated with different concentration of Textilinin-1 (0, 2, 4, 8 μM), monitored over 24 h by measuring OD600 (*n* = 8). (**C**) Representative images of LB agar plates after 24 h incubation showing colony formation under different treatments. Upper panel: *E. coli*; lower panel: *S. aureus*. From left to right: 0, 2, 4, 8 μM Textilinin-1. Bar graphs on the right quantify colony numbers (*n* = 4), reduced *S. aureus* colony formation after 8 μM Textilinin-1 treatment, while no significant reduction was observed for *E. coli*. Data are presented as mean ± SD. ** *p* < 0.01, ns: not significant.

Textilinin-1 exhibited potent and selective antibacterial activity, specifically targeting the Gram-positive pathogen *S. aureus* while sparing the Gram-negative *E. coli*. As shown in Figure 10A, Textilinin-1 showed no significant effect on the growth of *E. coli*. Over the 24 h, optical density measurements revealed similar growth kinetics across all treatment groups (Figure 10A), and the number of viable colonies at 24 h confirmed no reduction in viable bacteria compared to control (Figure 10C). Representative agar plates revealed comparable colony densities across groups, indicating lack of antibacterial activity against Gram-negative strains. In contrast, Textilinin-1 exhibited pronounced antibacterial activity against *S. aureus*. A marked delay in bacterial proliferation was as early as 4 h, with significantly reduced OD_600_ values by 24 h (*p* < 0.01; Figure 10B). This growth suppression effect was confirmed by colonies counting analysis, which showed a substantial decrease in viable colonies (*p* < 0.01; Figure 10C). Representative agar plates further exhibited both fewer and smaller colonies in the Textilinin-1-treated group, consistent with impaired bacterial replication and viability. Together, these results demonstrate that Textilinin-1 selectively inhibits the growth of Gram-positive *S. aureus* while no efficacy against Gram-negative *E. coli* under the same conditions.

In addition to the laboratory strain assays, a clinical isolate of *S. aureus* obtained from a diabetic foot ulcer sample was used to further evaluate the antibacterial potential of Textilinin-1. In this clinically relevant strain, the inhibitory profile showed a distinct time dependent pattern when compared with standard antibiotics. At 6 h, Textilinin-1 exhibited weaker suppression of bacterial growth relative to penicillin. However, a clear reversal occurred after 12 h. From 12 h onward, cultures treated with Textilinin-1 maintained nearly unchanged bacterial density, whereas the cultures exposed to penicillin demonstrated a marked increase in bacterial burden, indicating loss of antibiotic effectiveness over time (Figure S3, *p* < 0.0001). By 18 h, Textilinin-1 showed markedly superior antibacterial activity compared with penicillin. Notably, this clinical isolate displayed strong resistance to ciprofloxacin at 5 µg/mL, yet remained sensitive to Textilinin-1 (Figure S3, *p* < 0.0001). These findings further support the selective antibacterial activity of Textilinin-1 and its potential utility in infections caused by drug resistant *S. aureus*.

### 3.5. Protein-Protein Docking Predicts Potential Interactions Between Textilinin-1 and Key Wound Healing Membrane Receptors

Exploratory protein-protein docking simulations identified potential interactions between Textilinin-1 and selected wound healing-related receptors, providing preliminary hypotheses for future mechanistic validation. To explore potential molecular interactions that may warrant future mechanistic validation, an exploratory protein-protein docking was performed using the HDOCK server. Six receptors central to wound healing were selected, including PDGFRβ and VEGFR2 (tissue repair and angiogenesis), CCR2, CXCR2 and CXCR3 (leukocyte recruitment and effect angiogenic), and TLR4 (innate immune activation), to represent distinct categories of wound-healing-related biological functions.

Docking models suggested that Textilinin-1 is predicted to reside within or near the putative ligand-binding pockets of all six targets (Figure S4). In each complex, Textilinin-1 (yellow) was modelled within, or near, the predicted ligand-binding pocket of the target protein (brown). The spatial orientation of the ligand relative to key binding residues indicated favorable steric complementarity and the potential for stable intermolecular interactions. These qualitative predictions were consistent with the quantitative docking results summarized in Table S2.

Across all six targets, docking confidence scores exceeded 0.85, indicating that the generated docking poses were computationally plausible within the HDOCK framework. Binding affinity scores (docking scores) ranged from –238.56 for CXCR3 to –288.54 for CXCR2. However, several complexes, particularly CCR2 (155.15 Å) and CXCR2 (124.93 Å), exhibited markedly elevated RMSD values. Such high RMSD indicates substantial variation among multiple predicted binding poses, implying reduced conformational stability or increased flexibility within the binding site.

Together, these in silico findings should be interpreted as exploratory and hypothesis-generating, provide a theoretical basis suggesting that Textilinin-1 could potentially modulate multiple signalling receptors involved in inflammation, angiogenesis, and tissue regeneration in wound healing process.

## 4. Discussion

In the present study, we evaluated the preclinical effects of Textilinin-1 in wound repair models and observed treatment-associated anti-inflammatory, antibacterial, and pro-repair responses during wound repair. These effects should first be interpreted in relation to the established pharmacological profile of Textilinin-1 as a Kunitz-type serine protease inhibitor [15,37]. Plasmin and related protease systems are involved not only in fibrinolysis, but also in inflammatory regulation, extracellular matrix turnover, growth factor availability, cell migration, and tissue repair [15,37,38,39]. Plasmin and related trypsin-like proteases are known endogenous activators of protease-activated receptors (PARs), which are involved in inflammation and tissue repair [38,39,40,41]. Within this framework, the wound repair- and protease-responsive transcriptional changes observed in this study are interpreted as potential downstream consequences of modulating protease-PAR signalling, rather than as evidence of direct receptor binding by Textilinin-1.

Consistent with our bioinformatic predictions (Figure 1 and Figure 2), and the established role of macrophages in wound repair [42], Textilinin-1 attenuated M1-like inflammatory activation in LPS-stimulated macrophages, as shown by reduced the expression of M1-associated inflammatory markers (Cxcl1, Cxcl10, Tnf, and iNos) without affecting the M2 marker *Arg1* expression (Figure 9). From this protease-centred perspective, these effects may be consistent with the involvement of plasminogen/plasmin and trypsin-like serine proteases in inflammatory amplification, protease-activated receptor signalling, and wound repair [43]. This finding suggests that Textilinin-1 does not exert broad, non-specific immunosuppressive actions. Although inflammation is essential for debridement and pathogen clearance, excessive or prolonged inflammation is a primary cause of delayed healing and fibrosis [7,44,45]. In parallel, Textilinin-1 showed growth-inhibitory activity against *S. aureus* under the conditions tested (Figure 10 and Figure S3), a clinically relevant wound pathogen and contributor to diabetic foot ulcer pathology [46,47]. This finding aligns with our KEGG enrichment analysis, which revealed significant involvement of genes associated with the “*S. aureus* infection” pathway, suggesting a possible link between Textilinin-1-associated protease networks and host responses relevant to bacterial infection. The observed selective efficacy may reflect structural differences in bacterial cell walls, particularly the thick peptidoglycan layer of *S. aureus* [48]. However, the antibacterial findings remain preliminary. To properly assess the risk of future resistance development, future studies should elucidate the mechanism of action of Textilinin-1 and expand MIC (minimum inhibitory concentration) and MBC (minimum bactericidal concentration) evaluations across a broader panel of clinical isolates and biofilm-associated pathogens. Together, these findings suggest that Textilinin-1 may influence inflammatory and infection-related processes during wound repair [7,47,49,50].

Another salient finding is the strong pro-angiogenic capacity of Textilinin-1. It promoted endothelial cell proliferation, migration, and capillary-like network formation in vitro (Figure 3 and Figure 7), accompanied by upregulation of key angiogenic mediators such as Vegfc and its receptor Vegfr2 [51,52,53,54]. Re-establishing vascular supply is critical for delivering oxygen and nutrients to the regenerating tissue [55]. In addition to its anti-inflammation properties, these findings suggest that Textilinin-1 may support the proliferation phase of wound healing by stimulating the core cellular processes involved in tissue reconstruction [45,56]. During this re-vascularisation phase, Textilinin-1 also enhanced the proliferation and migration of both dermal fibroblasts (Figure 4) and epidermal keratinocytes (Figure 5), which are responsible for rebuilding the dermal matrix and restoring the skin’s barrier function, respectively [57,58,59]. These pro-migratory and pro-angiogenic responses may also be consistent with protease-associated regulation of extracellular matrix turnover, growth factor bioavailability, and tissue reconstruction during the proliferative phase of repair [38,60,61]. Moreover, Textilinin-1 appeared to modulate fibroblast ROS levels (Figure 8). While excessive ROS can damage cells and impair wound healing [62], moderate ROS production functions as a signaling cue to promote cell proliferation and migration [63,64]. Together, these ROS- and gene-expression findings suggest potential redox- and proliferation-related cellular responses to Textilinin-1.

These in vitro activities corresponded to accelerated wound closure and enhanced tissue quality in vivo. In both murine and porcine models, topical application of Textilinin-1 led to faster re-epithelialization and more organized granulation tissue formation (Figure 6 and Figure S2). This preliminary porcine data should be interpreted as proof-of-concept efficacy in a large animal model and support future studies with expanded cohorts to evaluate inter-animal variability. This reparative pattern is consistent with established benchmarks in the literature, where bioactive dressings have been shown to significantly improve granulation thickness and collagen maturity in similar full-thickness defect models [49,65]. Together, these findings suggest that Textilinin-1 support multiple cellular and tissue-level processes involved in wound repair, although direct comparisons with advanced wound-healing systems were not performed. Markedly improved epithelial thickness, collagen deposition, and dermal organization has observed, although the overall closure rate showed only modest improvement (Figure 6). Gene expression profiling revealed a time-dependent pattern. During the early healing phase (day 7 post injury with daily Textilinin-1 treatment), Textilinin-1 increased the pro-inflammatory cytokines IL-1β and TNF-α, which are necessary during the initial inflammatory response [66,67,68,69,70], and upregulated various pro-reparative genes. By day 14, these cytokines were markedly downregulated, in line with a decelerating and eventually resolving inflammatory response. This time-dependent pattern may reflect transient early inflammatory signalling followed by reduced inflammatory activity at a later repair stage. However, because earlier time points such as Days 1 and 3 were not assessed and these findings were measured at the transcript level, further time-course and protein-level validation are required to determine whether Textilinin-1 transiently enhances early inflammation, accelerates resolution, or shifts the inflammatory-to-proliferative transition.

In the remodeling phase, where the extracellular matrix (ECM) reorganization and scar maturation predominate, Textilinin-1 showed little direct effect. In NIH/3T3 fibroblasts, Textilinin-1 treatment did not significantly alter the expression of key remodeling-related genes, including MMPs, TIMPs, and collagens (Figure S1). This ostensibly “negative” finding is mechanistically informative, suggesting that Textilinin-1 primarily during the early and intermediate phases of healing, namely inflammation and proliferation, rather than directly driving late-stage matrix remodeling. Alternatively, this observation may reflect the limitations of our simplified in vitro monoculture system, which lacks the cellular diversity (e.g., macrophages, fibroblasts) and interplay, and ECM dynamics required to model the remodeling microenvironment [71,72,73,74,75,76]. Moreover, the 24 h treatment window may have been insufficient to capture remodeling-associated transcriptional changes, which typically evolve over weeks to months. Collectively, these data suggest that Textilinin-1 helps establish a favorable microenvironment for subsequent remodeling rather than directly mediating it.

Within this protease-centred framework, the docking analysis was included as an exploratory, hypothesis-generating approach to identify receptor-related interactions that may warrant future validation, rather than as evidence replacing the established protease-inhibitory framework. Our in silico docking analysis suggests that Textilinin-1 could potentially interact with multiple cell surface receptors involved in inflammation and repair, such as chemokine receptors (CCR2, CXCR2) and growth factor receptors (VEGFR2, PDGFRβ) (Figure S4, Table S2). All predicted complexes yielded confidence scores exceeding 0.9, indicating that the predicted poses were computationally plausible within the HDOCK framework, although these scores do not establish physiological binding or functional receptor engagement. Such predicted interactions could underlie activation of key downstream pathways such as PI3K/AKT, MAPK/ERK, chemokine, and TLR/NF-kB signaling [7,27,77,78]. However, whether Textilinin-1 directly engages these receptors or influences these pathways requires further experimental validation. Notably, the high RMSD values across key receptor docking predictions (Table S2) indicate substantial variability among predicted binding poses, suggesting uncertainty in these predicted interactions rather than confirming a stable binding mode [79,80]. In line with this, Textilinin-1 differently regulated Cxcl1 and Cxcl10, suppressing their expression in inflamed macrophages while inducing them in endothelial cells (Figure 7 and Figure 8). Further biophysical validation, such as surface plasmon resonance (SPR), isothermal titration calorimetry (ITC), PAR1/2 and PAR4 inhibitor experiments, receptor-blocking antibody approaches, assessment of Textilinin-1 cell-surface binding and internalization by confocal microscopy, and downstream phosphorylation or pathway inhibition analyses, will be required to determine whether receptor-mediated signalling contributes to the biological effects of Textilinin-1.

Several points should be considered when interpreting these findings. First, although the data suggest that Textilinin-1 is associated with inflammatory, proliferative, angiogenic, and antibacterial responses, these effects should be interpreted as treatment-associated responses rather than as confirmation of a direct molecular mechanism. Future studies incorporating inactive or mutant protein controls and protein-level pathway validation will help further define the downstream signalling pathways involved. Second, pharmacokinetic and formulation-related parameters, including bioavailability, stability, tissue penetration, and local retention after topical application, will be important for future dose optimization and clinical translation. Third, the in vivo models were acute excisional wounds in otherwise healthy animals, and the porcine model was conducted as a pilot study. Future studies in larger cohorts and in diabetic, infected, ischemic, aged, or otherwise compromised wound models are required to assess relevance to chronic wound repair. Finally, several transcript-level and histological findings remain exploratory and require further validation. Overall, the bioinformatics and docking analyses should be viewed as hypothesis-generating rather than as confirmed molecular explanations for the observed effects of Textilinin-1 during wound repair [77].

## 5. Conclusions

In summary, our present investigation provides preclinical evidence that Textilinin-1 treatment is associated with multiple wound repair-related responses, which may be interpreted in relation to its established haemostatic and serine protease inhibitory activity. The therapeutic potential of Textilinin-1 lies in the synergy between potent anti-inflammatory and pro-angiogenic effects, augmented by antimicrobial and broad pro-proliferative actions. Together, these findings support further investigation of Textilinin-1 as a candidate for cutaneous wound repair applications, with future studies needed to define its precise mechanisms, optimize dosing and delivery, and evaluate its efficacy in chronic or otherwise compromised wound models.

## Data Availability

All data generated or analyzed during this study are included in this published article and its Supplementary Material. Source data underlying the figures (including raw qPCR Ct values, wound area measurements, and bacterial growth and colony count data) are available from the corresponding author upon reasonable request.

## Supplementary Materials

The following supporting information can be downloaded at https://www.mdpi.com/article/10.3390/pharmaceutics18060762/s1: Figure S1: Effect of Textilinin-1 on extracellular matrix remodelling–related gene expression in NIH/3T3 fibroblasts; Figure S2: Textilinin-1 accelerates wound healing in a porcine excisional model; Figure S3: Textilinin-1 inhibits the proliferation of a clinical Staphylococcus aureus isolate; Figure S4. Protein-protein docking models of Textilinin-1 with membrane receptor targets involved in wound healing; Table S1: List of primers and sequences used for qPCR; Table S2. Docking scores, confidence scores, and ligand RMSD values for Textilinin-1 binding to selected protein targets as predicted by HDOCK.

## Abbreviations

The following abbreviations are used in this manuscript:

ARG1	Arginase-1
iNOS	Inducible nitric oxide synthase
LPS	Lipopolysaccharide
PBS	Phosphate-buffered saline
DMEM	Dulbecco’s modified eagle medium
FBS	Fetal bovine serum
KC-SFM	Keratinocyte serum-free medium
CCK-8	Cell counting kit-8
DCFH-DA	2′,7′-dichlorofluorescein diacetate
ROS	Reactive oxygen species
OD600	Optical density at 600 nm
qPCR	Quantitative polymerase chain reaction
MFI	Mean fluorescence intensity
PLG	Plasminogen
PRSS1	Serine protease 1 (trypsin-1)
PPI	Protein–protein interaction
GO	Gene ontology
KEGG	Kyoto encyclopedia of genes and genomes
STRING	Search tool for the retrieval of interacting genes/proteins
DAVID	Database for annotation, visualization, and integrated discovery
NFF	Normal human dermal fibroblasts
EHS	Engelbreth-Holm-Swarm (matrix extract)
EDTA	Ethylenediaminetetraacetic acid
PE	Phycoerythrin
APC	Allophycocyanin
