# Supplementary material for: Textilinin-1, a Snake Venom-Derived Kunitz-Type Protease Inhibitor, Accelerates Wound Healing Through Anti-Inflammatory, Antibacterial, and Pro-Regenerative Activities
Source: Pharmaceutics. 2026 Jun 22;18(6):762. doi: 10.3390/pharmaceutics18060762 (PMC13307457; doi:10.3390/pharmaceutics18060762)
Supplement: Supplementary file 1 [file pharmaceutics-18-00762-s001.zip › pharmaceutics-4285191-supplementary.pdf]

Supplementary materials

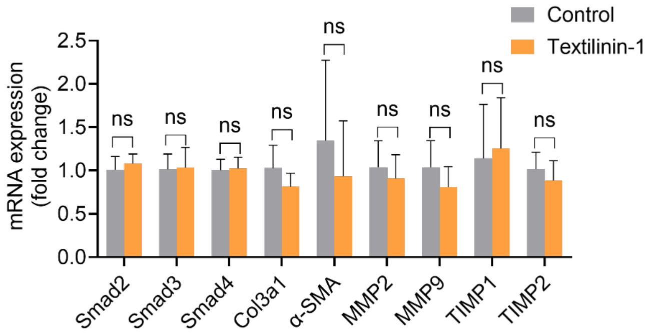

Figure S1. Effect of Textilinin-1 on extracellular matrix remodelling–related gene expression in NIH/3T3 fibroblasts. NIH/3T3 cells were treated with vehicle control or 8 μM Textilinin-1 for 24 h, and mRNA expression levels of extracellular matrix remodelling–associated genes were quantified by qPCR. Data are presented as mean ± SD (n = 3 independent experiments). No statistically significant differences ($p > 0.05$) were detected between control and Textilinin-1–treated groups for any of the analyzed genes (two-tailed unpaired Student's t-test).

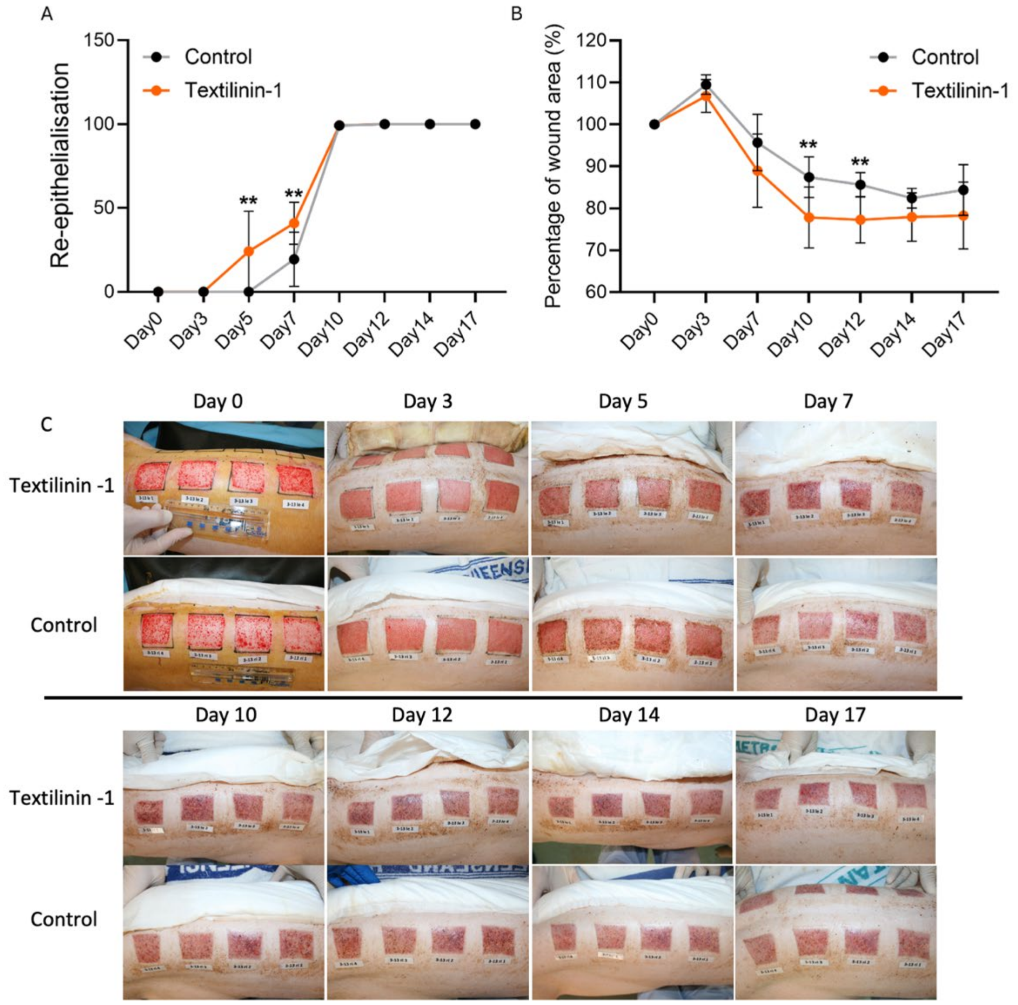

Figure S2. Textilinin-1 accelerates wound healing in a porcine excisional model. (A) Time course of re-epithelialization (%) in Textilinin-1–treated versus control wounds from day 0 to day 17. (B) Wound area expressed as a percentage of the baseline area (day 0) over time. (C) Representative macroscopic images of paired dorsal wounds at the indicated time points (Textilinin-1, left side; Control, right side). In this pilot study, Textilinin-1-treated wounds were placed on the left side and control wounds on the right side of one healthy adult pig. Data are presented as mean ± SD of wound-level measurements from one pilot animal and are intended for descriptive interpretation only. Multiple wounds from the same animal were not treated as independent biological replicates for definitive statistical inference.

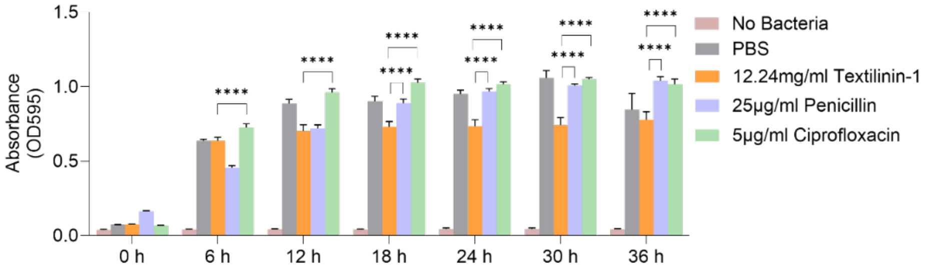

Figure S3. Textilinin-1 inhibits the proliferation of a clinical Staphylococcus aureus isolate. Growth curves of a clinical Staphylococcus aureus isolate cultured in the presence of PBS, 12.4mg/ml Textilinin-1, 25µg/ml Penicillin, 5µg/ml Ciprofloxacin. Bacterial proliferation was monitored by measuring OD595 at 0, 6, 12, 18, 24, 30 and 36 hours. Data are presented as mean ± SD (n=7). ****p < 0.0001.

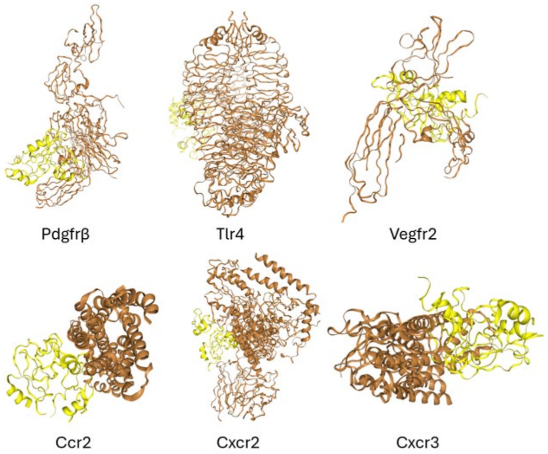

Figure S4. Protein-protein docking models of Textilinin-1 with membrane receptor targets involved in wound healing. Representative docking models showing the predicted binding conformations between Textilinin-1 (ligand, yellow) and key membrane protein receptors (brown), including PDGFRβ, TLR4, VEGFR2, CCR2, CXCR2, and CXCR3. These receptor targets were identified through an integrative pipeline combining bioinformatics prediction, differential gene expression analysis from cell and animal experiments, and pathway enrichment analyses. Specifically, upregulated or downregulated genes involved in wound healing–related pathways were mapped to corresponding membrane receptors, which were then selected for molecular docking analysis to evaluate potential interactions with Textilinin-1.

Table S1. List of primers and sequences used for qPCR.

Gene	Primer	Sequence
CyclinD1	Forward	5′-TCAAGTGTGACCCGGACTG-3′
	Reverse	5′-TCTTCCTCCACTTCCCCCTC-3′
Cdk2	Forward	5′-CCTAGTGTGTACCCAGCACC-3′
	Reverse	5′-TCCTTGTGATGCAGCCACTT-3′
Pdgf	Forward	5′-AATGCTGAGCGACCACTCCATC-3′
	Reverse	5′-TCGGGTCATGTTCAAGTCCAGC-3′
Bcl-2	Forward	5′-GGGGTCATGTGTGTGGAGAG-3′
	Reverse	5′-AGGGTCTTCAGAGACAGCCA-3′
Atg13	Forward	5′-TCCTATCAGCCTGCTGTCCT-3′
	Reverse	5′-AGAAGGCATGTGCATCACCA-3′
Erk1	Forward	5′-CCCATCCCAGGAGGACCTTA-3′
	Reverse	5′-TGATGCGCTTGTTTGGGTTG-3′
Erk2	Forward	5′-AAATTGGTCAGGACAAGGGCTC-3′
	Reverse	5′-GTCAAGAGTGGGTAAGCTGAGA-3′
Smad7	Forward	5′-GCATTCCTCGGAAGTCAAGA-3′
	Reverse	5′-CAGGCTCCAGAAGAAGTTGG-3′
Dhcr24	Forward	5′-GAGCCCTTGGTGTCTATGGG-3′
	Reverse	5′-GACGATGACTCGATGCCTGT-3′
Dnaja2	Forward	5′-AGGATTACGTTCACTGGGGA-3′
	Reverse	5′-TCCCATCTCTCTGGAACACC-3′
Foxo1	Forward	5′-CGGAAAATCACCCCGGAGAA-3′
	Reverse	5′-GAGCTGGTTCGAGGACGAAA-3′
Vegfc	Forward	5′-TCTGTGTCCAGCGTAGATGAG-3′
	Reverse	5′-GTCCCCTGTCCTGGTATTGAG-3′
Vegfd	Forward	5′-CCTATTGACATGCTGTGGGAT-3′
	Reverse	5′-GTGGGTTCCTGGAGGTAAGAG-3′
Vegfr2	Forward	5′-CTGCCTACCTCACCTGTTTCC-3′
	Reverse	5′-CGGCTCTTTCGCTTACTGTTC-3′
Ccl2	Forward	5′-CCTGCTGTTCACAGTTGCC-3′
	Reverse	5′-ATTGGGATCATCTTGCTGGT-3′
Cxcl1	Forward	5′-CCACACTCAAGAATGGTCGC-3′
	Reverse	5′-TCTCCGTTACTTGGGGACAC-3′

Cxcl10	Forward	5′-CTCATCCTGCTGGGTCTGAG-3′
	Reverse	5′-CCTATGGCCCTCATTCTCAC-3′
iNos	Forward	5′-GTTCTCAGCCCAACAATACAAGA-3′
	Reverse	5′-GTGGACGGGTCGATGTCAC-3′
Tnf	Forward	5′-GGTGCCTATGTCTCAGCCTCTT-3′
	Reverse	5′-GCCATAGAACTGATGAGAGGGAG-3′
Il-1β	Forward	5′-TGGACCTTCCAGGATGAGGACA-3′
	Reverse	5′-GTTCATCTCGGAGCCTGTAGTG-3′
Tgfβ	Forward	5′-TGATACGCCTGAGTGGCTGTCT-3′
	Reverse	5′-CACAAGAGCAGTGAGCGCTGAA-3′
Vegf	Forward	5′-CTGCTGTAACGATGAAGCCCTG-3′
	Reverse	5′-GCTGTAGGAAGCTCATCTCTCC-3′
Col1a1	Forward	5′-CCTCAGGGTATTGCTGGACAAC-3′
	Reverse	5′-CAGAAGGACCTTGTTTGCCAGG-3′
Col3a1	Forward	5′-GACCAAAAGGTGATGCTGGACAG-3′
	Reverse	5′-CAAGACCTCGTGCTCCAGTTAG-3′
Actb	Forward	5′-ACCTTCTACAATGAGCTGCG-3′
	Reverse	5′-CTGGATGGCTACGTACATGG-3′

Table S2. Docking scores, confidence scores, and ligand RMSD values for Textilinin-1 binding to selected protein targets as predicted by HDOCK.

**Protein**	**Input PDB ID**	**Docking Score**	**Confidence Score**	**Ligand RMSD (Å)**
**Pdgfrβ**	3MJG	-272.84	0.9211	88.00
**Tlr4**	3FXI	-267.50	0.9129	47.64
**Vegfr2**	3V2A	-264.71	0.9084	44.35
**Ccr2**	5T1A	-286.59	0.9389	155.15
**Cxcr2**	6LFO	-290.69	0.9434	209.23
**Cxcr3**	8HNN	-271.32	0.9188	141.77
